# Quantifying hepatic arterial blood flow with 4D flow MRI: an in vivo feasibility and repeatability study

**DOI:** 10.1007/s10334-026-01331-0

**Published:** 2026-02-14

**Authors:** Anne van den Brekel, Marnix C. Maas, Ilse M. Spenkelink, Willem C. M. van Tilburg, Joey Roosen, Daniel Giese, Ning Jin, Jurgen J. Fütterer, J. Frank W. Nijsen

**Affiliations:** 1 Department of Medical Imaging, Radboud University Medical Center, Nijmegen, The Netherlands; 2 Research and Clinical Translation, Magnetic Resonance, Siemens Healthineers AG, Erlangen, Germany; 3 MR R&D, Siemens Healthineers USA, Malvern, PA USA

**Keywords:** Magnetic resonance imaging, Flow quantification, 4D flow MRI, Phase-contrast MRI, Hepatic arterial blood flow

## Abstract

**Objective:**

To investigate the feasibility and repeatability of four-dimensional (4D) flow MRI for quantifying hepatic arterial blood flow (HABF) in healthy volunteers and patients with liver disease, and to identify sources of measurement variability.

**Materials and methods:**

In this prospective single-centre study, 23 healthy volunteers and 12 patients underwent two-dimensional (2D) and 4D flow acquisitions at 3T. Time-averaged and peak systolic vessel areas, velocities, and flow rates of the common hepatic artery (CHA) were extracted. Feasibility of 4D flow was assessed via CHA visibility on phase-contrast MRA. Scan-rescan repeatability of 4D flow was assessed by calculating within-subject coefficient of variation (CV_within_) using two-level variance components analysis (VCA). Within-subject variance was further decomposed into acquisition-related and plane positioning components using three-level VCA. Agreement between 2D and 4D flow was evaluated using Bland–Altman analysis and Pearson’s correlation.

**Results:**

Average 4D flow feasibility rate was 83%. CV_within_ ranged from 12–30%, with acquisition-related factors and plane positioning accounting for 30–40% and 7–11%, respectively. 4D flow overestimated vessel area by ~ 18% and underestimated velocity and flow rate by ~ 53% and ~ 36%, respectively, compared to 2D flow.

**Discussion:**

4D flow MRI enables HABF quantification, though substantial scan-rescan variability exists. Acquisition-related factors were the main source of within-subject variability, while plane positioning contributed less.

**Supplementary Information:**

The online version contains supplementary material available at https://doi.org/10.1007/s10334-026-01331-0.

## Introduction

Transarterial radioembolisation (TARE) is a locoregional treatment for hepatocellular carcinoma (HCC) and liver metastases [1], involving intra-arterial injection of radioactive microspheres into hepatic arterial branches supplying the tumour(s). TARE exploits the liver’s dual blood supply, with normal liver tissue receiving 70–80% from the portal vein and 20–30% from the hepatic artery, while tumours rely almost exclusively on arterial perfusion [2].

Delivering a high radiation dose to the tumour(s) is critical, as higher tumour-absorbed doses correlate with improved survival [3, 4]. Simultaneously, limiting the dose to healthy liver tissue is essential to prevent toxicity. However, predicting or controlling microsphere distribution remains challenging. Factors such as catheter position, injection velocity, tumour burden, and hepatic arterial blood flow (HABF) may influence the distribution [5].

Our recent study in perfused pig livers showed that increased HABF rates led to more homogeneous microsphere distributions compared to lower rates [6], warranting the need for further investigation. However, an accurate, precise, and non-invasive clinically accepted method to quantify HABF in vivo is currently lacking.

Three-dimensional, time-resolved phase-contrast MRI with three-dimensional velocity encoding (4D flow MRI) is a promising technique for measuring HABF. It is currently used clinically to assess flow in the heart and great vessels [7]. Compared to two-dimensional, time-resolved phase-contrast MRI with through-plane velocity encoding (2D flow MRI) and Doppler ultrasound, it allows retrospective plane positioning.

Accurate plane positioning is essential for reliable flow measurements [8], but might be complex in the hepatic artery due to anatomical variations, tortuosity, respiratory motion, and small vessel size. The latter is likely particularly challenging, as 5–6 voxels across the vessel diameter are needed for accurate flow quantification [9].

In this study, we investigated the feasibility and repeatability of 4D flow MRI for quantifying HABF in healthy volunteers and patients with liver disease (cirrhosis and/or malignancy), and explored sources of measurement variability. Building on the work of Dimov et al. [10], who studied spatial resolution and respiratory gating effects in vitro and in healthy volunteers, we used a higher spatial resolution, evaluated scan-rescan repeatability, and extended the application to patients.

## Materials and methods

### Subjects

This study was approved by the Medical Research Ethics Committee Oost-Nederland (reference NL58944.091.16, 2016–2774, project 23,047/24009), ensuring compliance with ethical standards for human research as outlined in the Declaration of Helsinki. Written informed consent was obtained from all subjects. Thirty-five legally competent adults were prospectively recruited in three subgroups between November 2023 and September 2024. Subgroup 1 consisted of eleven volunteers aged between 18 and 30 years with no history of liver disease. Subgroup 2 comprised twelve volunteers aged 55 years or older with no history of liver disease. Subgroup 3 consisted of twelve patients with a (suspected) liver disease (cirrhosis and/or malignancy), scheduled for a clinical liver MRI with intravenous contrast (DOTAREM® 0.5 mmol/mL, Guerbet, Villepinte, France) as standard-of-care. Subgroup 2 served as an age-matched control group for patients. Volunteers were recruited via the institutional volunteer database and flyers. Patients were asked to participate via a telephone call by a researcher prior to their scheduled clinical liver MRI. Study information was sent via post or email to all subjects who expressed interest in participating. Informed consent was obtained prior to the MRI, either in advance or on the day of scanning. In participating patients, the study-specific MRI was performed immediately following the clinical liver MRI without repositioning. Exclusion criteria for all subgroups included MRI contraindications, claustrophobia, inability to provide informed consent, and presence of metal implants that could compromise image quality in the liver region.

### MRI acquisition

Subjects fasted for at least two hours before imaging on a 3T clinical MRI system (MAGNETOM Prisma Fit, Siemens Healthineers, Forchheim, Germany) using an 18-channel body coil and 32-channel spine coil. In volunteers, 3D T1-weigted gradient echo with Dixon fat separation, 2D T2-weighted steady-state free precession, and 3D T2*-weighted radial [11] sequences without contrast agent were acquired to locate the common hepatic artery (CHA). In patients, contrast-enhanced 3D T1-weigted gradient echo with Dixon fat separation and 2D T2-weighted steady-state free precession sequences from the same imaging sessions were used for CHA localisation.

In accordance with the 4D Flow MRI consensus statement [9, 12], the upper velocity encoding limit (VENC) was selected close to the expected maximum velocity (within 25% above) to optimise velocity-to-noise ratio and minimise aliasing. The VENC was determined via 2D flow scout sequences with a scan plane perpendicular to the CHA. After selecting the appropriate VENC, a final 2D flow sequence with an in-plane resolution matching the following 4D flow sequence was acquired. All 2D flow sequences were acquired in breath-hold with retrospective cardiac gating (electrocardiogram or pulse oximeter). Finally, a research compressed sensing (CS) 4D flow sequence was acquired twice in each volunteer without repositioning, and once in each patient. Acquisition parameters included retrospective cardiac gating (electrocardiogram or pulse oximeter), respiratory navigator gating (crossed pair navigator on liver dome, ± 6 mm window), CS acceleration factor of 7.6, and transverse orientation. Imaging parameters are listed in Table 1 and were based on published guideline recommendations [9, 12]. All imaging data were anonymised and stored in a secure research archive.

**Table 1:** MR imaging parameters

Sequence type	Acquired spatial resolution [mm^3^]	Reconstructed spatial resolution [mm^3^]	TE [ms]	TR [ms]	Temporal resolution [ms]	Flip angle [°]	Field of view [mm^2^]	Respiratory management	Cardiac phases	TA [min:sec]
3D T1-weighted gradient echo (Dixon)	1.20 × 1.20 × 6.00	1.20 × 1.20 × 3.00	1.29/2.52	3.97	NA	9	309 × 380	Breath hold (expiration)	NA	00:13
2D T2-weighted steady-state free precession	1.20 × 1.20 × 3.00	1.20 × 1.20 × 3.00	1.87	3.70	NA	41	311 × 380	Breath hold (expiration)	NA	00:19
3D T2*-weighted radial	1.30 × 1.30 × 2.60	1.30 × 1.30 × 1.30	6.00/12.00	15.20	NA	9	334 × 334	Free breathing	NA	08:22
2D flow VENC scout	1.77 × 1.77 × 6.00	1.77 × 1.77 × 6.00	2.47–3.35^†^	4.60–5.50^†^	37.12–44.16^†^	20	340 × 234	Breath hold (expiration)	30	00:15 ± 0:02
2D flow	1.25 × 1.25 × 6.00	1.25 × 1.25 × 6.00	2.77–3.10^†^	5.00–5.30^†^	40.16–42.80^†^	20	340 × 230	Breath hold (expiration)	30	00:21 ± 0:03
4D flow	1.25 × 1.25 × 1.25	1.25 × 1.25 × 1.25	2.73–3.23^†^	5.50–6.00^†^	43.68–47.68^†^	7	200 × 200	Navigator gating	14–31^‡^	12:57 ± 2:51

*TE* echo time, *TR* repetition time, *TA* acquisition time, *VENC* velocity encoding limit

^†^depending on VENC

^‡^depending on heart cycle

### Flow analysis

Flow analysis was performed by a technical physician with experience in 4D flow MRI acquisition and post-processing, under the supervision of a radiology resident trained in hepatic imaging. Both observers were familiar with the software and analysis protocol prior to the study. 2D flow datasets were analysed using Segment v4.1.0.1, R14284b (Medviso AB, Lund, Sweden; segment.heiberg.se) [13]. Background phase and anti-aliasing corrections were applied using the software’s default settings. All corrected datasets were visually inspected for residual artefacts.

Semi-automatic region of interest and vessel-tracking tools were used to segment the CHA lumen across cardiac phases. The following hemodynamic parameters were extracted: time-averaged area (area_avg_) [mm^2^], peak systolic area (area_peak_) [mm^2^], time-averaged velocity (v_avg_) [cm/s], peak systolic velocity (v_peak_) [cm/s], time-averaged flow rate (flow_avg_) [mL/s], and peak systolic flow rate (flow_peak_) [mL/s]. These parameters were selected based on prior literature. Peak systole was defined as the cardiac phase with the highest CHA velocity.

4D flow datasets were analysed using a prototype software (4D Flow Demonstrator Version 2.4, Siemens Healthineers, Forchheim, Germany). Background phase and anti-aliasing corrections were applied using default settings, and corrected datasets were visually inspected for residual artefacts. CHA segmentation was performed by manually placing seed points in the time-averaged phase-contrast magnetic resonance angiogram (PC-MRA), followed by automatic centerline and lumen extraction. Three analysis planes were positioned perpendicularly to the CHA segmentation, spaced at least 2.5 mm apart and positioned at the greatest possible distance from bifurcations. Hemodynamic parameters were extracted from each analysis plane using the same definitions as for 2D flow.

### Statistical analysis

Statistical analysis was conducted in SPSS v29.0 (IBM Corp., Armonk, NY, USA), with significance set at *p* < 0.05. Normality was assessed using the Shapiro–Wilk test. Parameters were expressed as mean ± standard deviation (SD) when normally distributed, or median with interquartile range when non-normally distributed.

Feasibility of 4D flow MRI was assessed based on image quality by the same researcher who performed the flow analysis. Image quality was considered sufficient when the CHA lumen was clearly visible on the PC-MRA, allowing successful segmentation and placement of analysis planes. This assessment was performed visually and classified using a binary yes/no decision, reflecting whether the vessel was adequately delineated for quantitative analysis.

Scan-rescan repeatability of 4D flow-derived hemodynamic parameters was assessed by quantifying within-subject standard deviation (SD_within_) and coefficient of variation (CV_within_) [14] using a variance components analysis (VCA), in which total variance in the most proximal plane was decomposed into between-subject and within-subject (i.e. scan-rescan) components. This was implemented using a linear random-effects model with ‘subject’ as a random intercept, estimated via restricted maximum likelihood (REML). SD, CV, and intraclass correlation coefficient (ICC) were calculated for total, between-subject, and within-subject variance components. Agreement between scan and rescan was visualised using Bland–Altman plots, with limits of agreement (LOA) defined as bias ± 1.96 × SD.

Influence of analysis plane positioning was evaluated using a three-level VCA across all three planes. Random-effects models included ‘subject’ and ‘acquisition’ as random intercepts, with ‘acquisitions’ nested within ‘subject’, yielding variance components for between-subject, within-subject due to acquisition, and within-subject due to plane positioning. SD, CV, and ICC were computed for each variance source to quantify their relative contributions.

Agreement between hemodynamic parameters derived from 2D flow and the most proximal 4D flow plane was assessed using Pearson’s correlation and Bland–Altman analysis, with LOA defined as bias ± 1.96 × SD.

Exploratory subgroup comparisons of 4D flow-derived hemodynamic parameters (acquisition 1, proximal plane) were performed using multiple pairwise independent samples *t*-tests (normally distributed data) or Mann–Whitney *U* tests (non-normally distributed data). Results were visualised using boxplots. No correction for multiple testing was applied due to the exploratory nature of the analysis.

## Results

### Subjects

The eleven and twelve volunteers included in subgroups 1 and 2, respectively, resulted in ten volunteers with 4D flow acquisitions of sufficient image quality in each subgroup. The twelve included patients yielded nine patients with 4D flow acquisitions of sufficient image quality. In the remaining volunteer from subgroup 1, the CHA could not be identified on anatomical scans due to severe intestinal gas, and therefore 2D and 4D flow sequences were not acquired. In the other remaining subjects, the CHA could not be located on PC-MRA due artifacts from cardiac (n = 4) or respiratory gating errors (n = 1). The 29 subjects (n = 10 in volunteer subgroup 1, n = 10 in volunteer subgroup 2, and n = 9 patients) with 4D flow acquisitions of sufficient image quality were included for further analyses. Their characteristics, 4D flow acquisition parameters, and 4D flow derived hemodynamic parameters are reported in Table 2. Additional patient characteristics are reported in Supplementary Table S1.

**Table 2:** Characteristics, 4D flow acquisition parameters and hemodynamic parameters of the two volunteer subgroups and patients with 4D flow acquisitions of sufficient image quality. Hemodynamic parameters are expressed as mean ± standard deviation (SD) when normally distributed, and median with interquartile range when not normally distributed. The range (minimum–maximum) was reported for each parameter as well

		Volunteer subgroup 1	Volunteer subgroup 2	Patients
Characteristics	Number of subjects	10	10	9
	Age (mean ± SD) [years]	26.9 ± 2.1	67.7 ± 9.1	61.4 ± 17.5
	Female [%]	50	50	22.2
	BMI (mean ± SD) [kg/m^2^]	22.1 ± 1.8	24.2 ± 2.9	26.2 ± 5.6
4D flow acquisition parameters	VENC (mean ± SD) [cm/s]	68.0 ± 9.2	77.0 ± 19.5	68.9 ± 13.6
	Cardiac phases (mean ± SD)	22.9 ± 4.0	21.5 ± 2.8	19.4 ± 2.8
	Temporal resolution (min–max) [ms]	45.0–47.7	43.7–47.7	44.5–47.7
	TA (mean ± SD) [min:sec]	13:56 ± 03:14	12:22 ± 02:25	11:40 ± 02:48
4D flow hemodynamic parameters	area_avg_ [mm^2^]	33.1 ± 6.7^a^; 24.8–47.0	26.0 ± 4.1^a,b^; 19.1–32.9	33.6 (21.1)^b^; 23.1–76.4
	area_peak_ [mm^2^]	32.4 ± 7.8^c^; 21.9–48.5	26.2 ± 4.4^c,d^; 19.4–33.2	31.1 (22.6)^d^; 22.1–72.5
	v_avg_ [cm/s]	9.5 ± 2.9; 5.8–13.9	11.5 ± 2.8; 6.9–16.3	8.8 ± 2.7; 5.5–13.3
	v_peak_ [cm/s]	20.5 ± 6.3; 11.9–34.8	22.6 ± 6.2; 13.9–34.8	21.4 ± 7.4; 9.4–31.6
	flow_avg_ [mL/s]	3.1 ± 1.2; 1.4–4.4	3.0 ± 0.9; 1.4–4.4	3.3 ± 1.0; 1.4–-4.7
	flow_peak_ [mL/s]	6.5 ± 2.2; 3.6–11.0	6.0 ± 2.1; 2.7–8.9	7.5 ± 1.7; 5.2–9.8

*SD* standard deviation, *BMI* body mass index, *VENC* velocity encoding limit, *TA* acquisition time, *area*_*avg*_ time-averaged area, *area*_*peak*_ peak systolic area, *v*_*avg*_ time-averaged velocity, *v*_*peak*_ peak systolic velocity, *flow*_*avg*_ time-averaged flow rate, *flow*_*peak*_ peak systolic flow rate

^a^significant difference, *p* = 0.01

^b^significant difference, *p* = 0.01

^c^significant difference, *p* = 0.04

^d^significant difference, *p* = 0.02

### Flow analysis

**Fig. 1:**
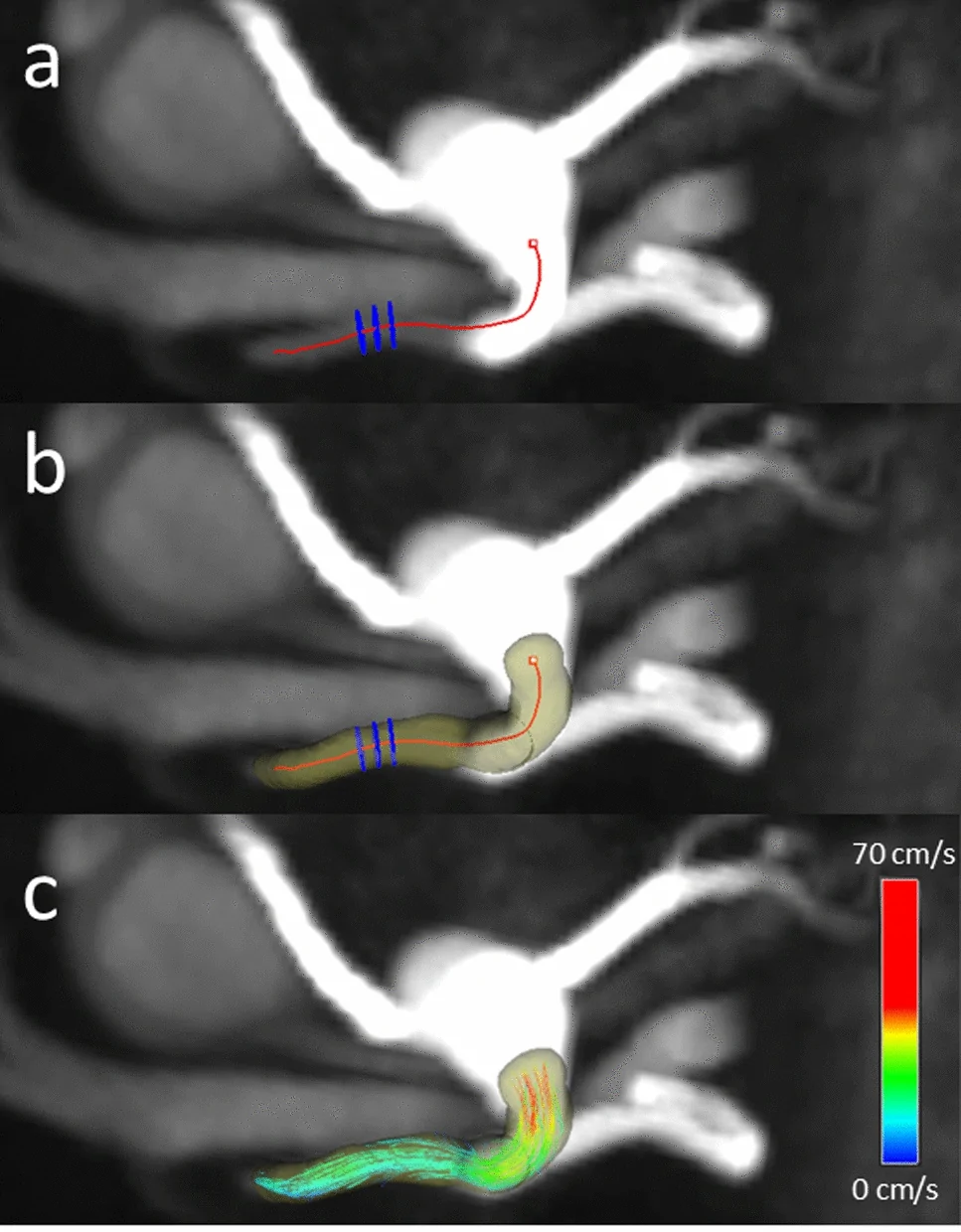
Time-averaged phase-contrast magnetic resonance angiogram with (**a**) vessel centerline (red) and analysis planes in the common hepatic artery (blue), **b** vessel centerline (red), analysis planes in the common hepatic artery (blue), and segmentation (yellow), and **c** segmentation (yellow) and streamlines visualising blood flow

**Fig. 2:**
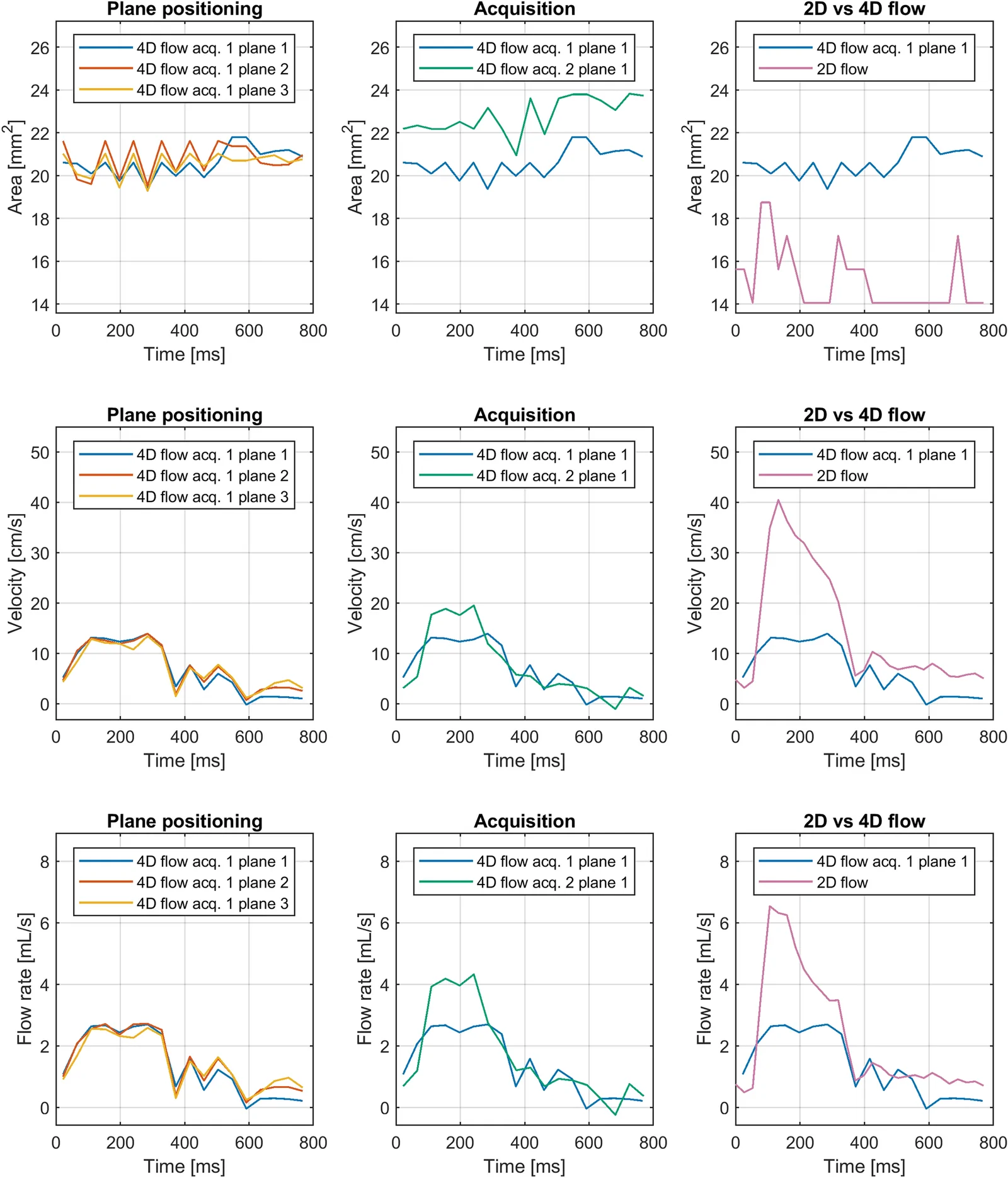
Time-resolved curves of area (top row), velocity (middle row), and flow rate (bottom row) of the common hepatic artery of one representative subject (subject 1 from volunteer subgroup 2). The left column shows data from the three analysis planes within the first 4D flow acquisition. The middle column compares the most distal plane from the first 4D flow acquisition with the most distal plane from the second 4D flow acquisition. The right column compares the 2D flow data with the most distal plane from the first 4D flow acquisition. Encoding velocity was 90 cm/s for both the 2D flow and 4D flow acquisition. The number of cardiac phases was 30 for the 2D flow and 19 for the 4D flow acquisition. *Acq* acquisition

An example PC-MRA with analysis planes in the CHA is shown in Fig. 1. The average number of voxels per vessel diameter was 5, with 20 voxels covering the vessel area. Time-resolved area, velocity, and flow rate curves are shown in Fig. 2 for one representative patient. Average analysis time was approximately 10 min per 2D flow dataset and 20 min per 4D flow dataset.

### Statistical analysis

Feasibility rates were 90.9%, 83.3%, and 75.0% in volunteer subgroup 1, volunteer subgroup 2, and the patient subgroup, respectively.

**Fig. 3:**
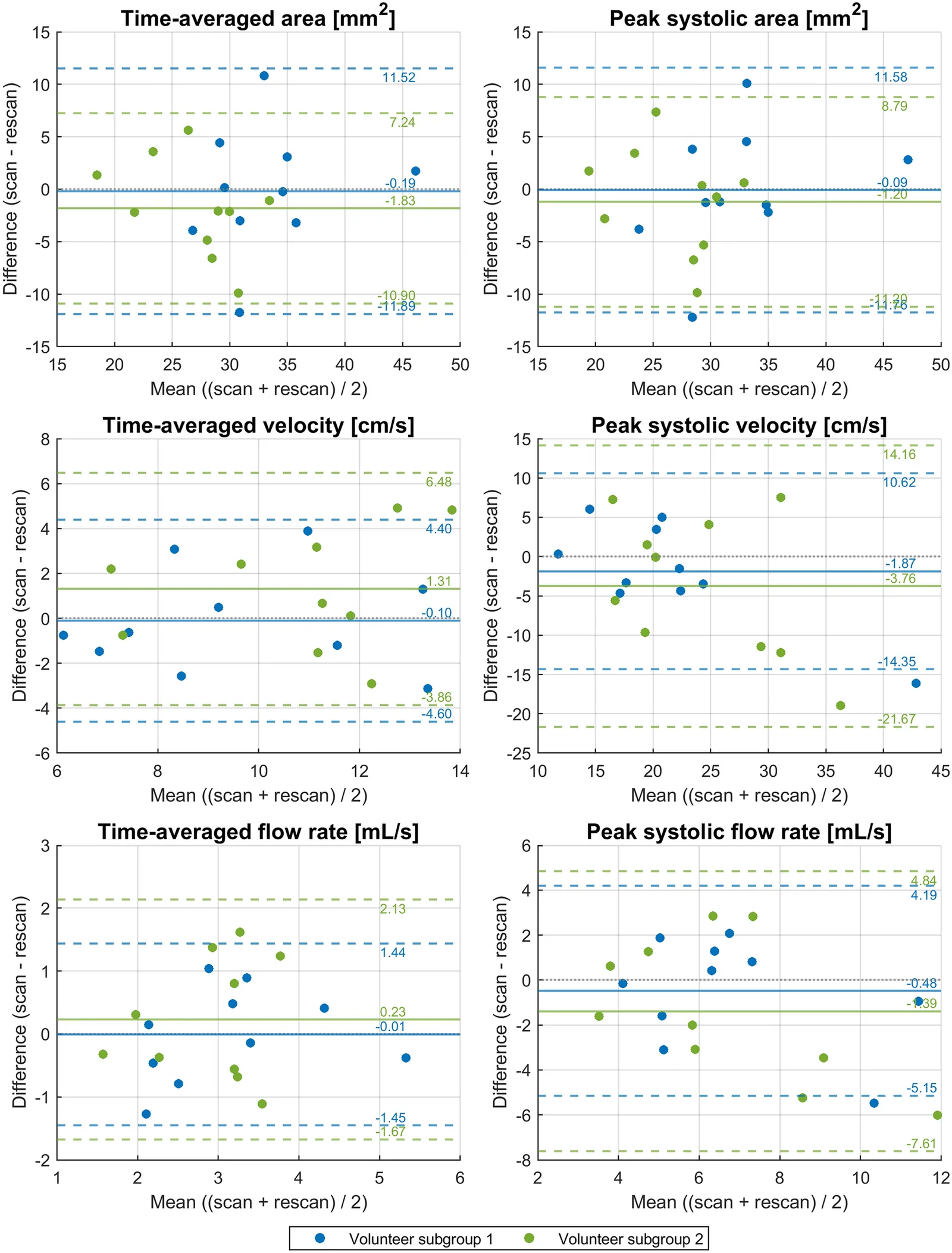
Bland–Altman plots of the hemodynamic parameters derived from the first and second 4D flow MRI acquisition. The dotted grey lines represent the zero-difference lines (y = 0); solid lines indicate the mean difference, and dashed lines represent the limits of agreement

Repeatability of 4D flow-derived hemodynamic parameters was evaluated using scan-rescan data from volunteers. Results are summarised in Table 3. CV_within_ ranged from 12.3 to 30.0%. Time-averaged parameters demonstrated better repeatability than peak systolic parameters, as reflected by lower CV_within_. Specifically, CV_within_ was slightly lower for area (area_avg_ 12.3% versus area_peak_ 12.7%), and substantially lower for velocity (v_avg_ 17.5% versus v_peak_ 24.8%) and flow (flow_avg_ 19.6% versus flow_peak_ 30.0%). Between-subject variance contributed substantially to total variance across all hemodynamic parameters, with ICC_between_ ranging from 48.4% for flow_peak_ to 66.9% for area_peak_. Within-subject variability accounted for the remaining 33.1 to 51.6% of the total variance. Scan-rescan agreement is visualised using Bland–Altman plots in Fig. 3. Overall bias and LOA are reported in Table 3.

**Table 3:** Repeatability metrics of 4D flow MRI-derived hemodynamic parameters from the most proximal analysis plane of acquisition 1 versus acquisition 2 of the volunteers

	Mean difference	Lower LOA	Upper LOA	SD_total_	CV_total_	SD_within_	CV_within_	ICC_between_	ICC_within_
area_avg_	− 1.0 mm^2^	− 11.3 mm^2^	9.3 mm^2^	6.4 mm^2^	21.2%	3.7 mm^2^	12.3%	66.3%	33.7%
area_peak_	− 0.6 mm^2^	− 11.3 mm^2^	10.0 mm^2^	6.5 mm^2^	22.1%	3.8 mm^2^	12.7%	66.9%	33.1%
v_avg_	0.6 cm/s	− 4.3 cm/s	5.5 cm/s	2.7 cm/s	26.9%	1.8 cm/s	17.5%	57.8%	42.2%
v_peak_	− 2.8 cm/s	− 18.0 cm/s	12.3 cm/s	8.7 cm/s	38.0%	5.7 cm/s	24.8%	57.5%	42.5%
flow_avg_	0.1 mL/s	− 1.6 mL/s	1.8 mL/s	1.0 mL/s	32.2%	0.6 mL/s	19.6%	63.2%	36.8%
flow_peak_	− 0.9 mL/s	− 6.4 mL/s	4.5 mL/s	2.8 mL/s	41.7%	2.0 mL/s	30.0%	48.4%	51.6%

*LOA* limit of agreement (calculated as bias ± 1.96 × standard deviation), *SD*_*total*_ total standard deviation, *CV*_*total*_ total coefficient of variation, *SD*_*within*_ within-subject standard deviation, *CV*_*within*_ within-subject coefficient of variation, *ICC*_*between*_ intraclass correlation coefficient for between-subject variance, *ICC*_*within*_ intraclass correlation coefficient for within-subject variance, *area*_*avg*_ time-averaged area, *area*_*peak*_ peak systolic area, *v*_*avg*_ time-averaged velocity, *v*_*peak*_ peak systolic velocity, *flow*_*avg*_ time-averaged flow rate, *flow*_*peak*_ peak systolic flow rate

To further identify the sources of within-subject variability, a three-level VCA was performed across all three measurement planes (Table 4). Acquisition-related variance was the largest contributor to within-subject variation (30.4 to 40.2% across parameters), while plane positioning contributed a smaller portion (6.8 to 11.4% across parameters). Combined, acquisition and plane positioning effects accounted for 38.5 to 51.6% of the total variance. Peak parameters again showed greater variability than time-averaged counterparts.

**Table 4:** Variance decomposition of 4D flow MRI-derived hemodynamic parameters across acquisitions and analysis planes

	SD_total_	CV_total_	ICC_between_	ICC_within, acquisition_	ICC_within, plane_	ICC_within, overall_
area_avg_	6.4 mm^2^	21.6%	61.5%	31.4%	7.1%	38.5%
area_peak_	6.5 mm^2^	22.0%	59.1%	34.1%	6.8%	40.9%
v_avg_	2.9 cm/s	27.6%	52.7%	38.2%	9.2%	47.3%
v_peak_	9.3 cm/s	40.5%	58.6%	30.4%	11.0%	41.4%
flow_avg_	1.0 mL/s	32.0%	58.1%	34.2%	7.6%	41.9%
flow_peak_	2.8 mL/s	42.3%	48.4%	40.2%	11.4%	51.6%

*SD*_*total*_ total standard deviation, *CV*_*total*_ total coefficient of variation, *ICC*_*between*_ intraclass correlation coefficient for between-subject variance, *ICC* intraclass correlation coefficient for within-subject variance due to acquisition, *ICC* intraclass correlation coefficient for within-subject variance due to plane positioning, *ICC* combined within-subject intraclass correlation coefficient (acquisition + plane), *area*_*avg*_ time-averaged area, *area*_*peak*_ peak systolic area, *v*_*avg*_ time-averaged velocity, *v*_*peak*_ peak systolic velocity, *flow*_*avg*_ time-averaged flow rate, *flow*_*peak*_ peak systolic flow rate

**Fig. 4:**
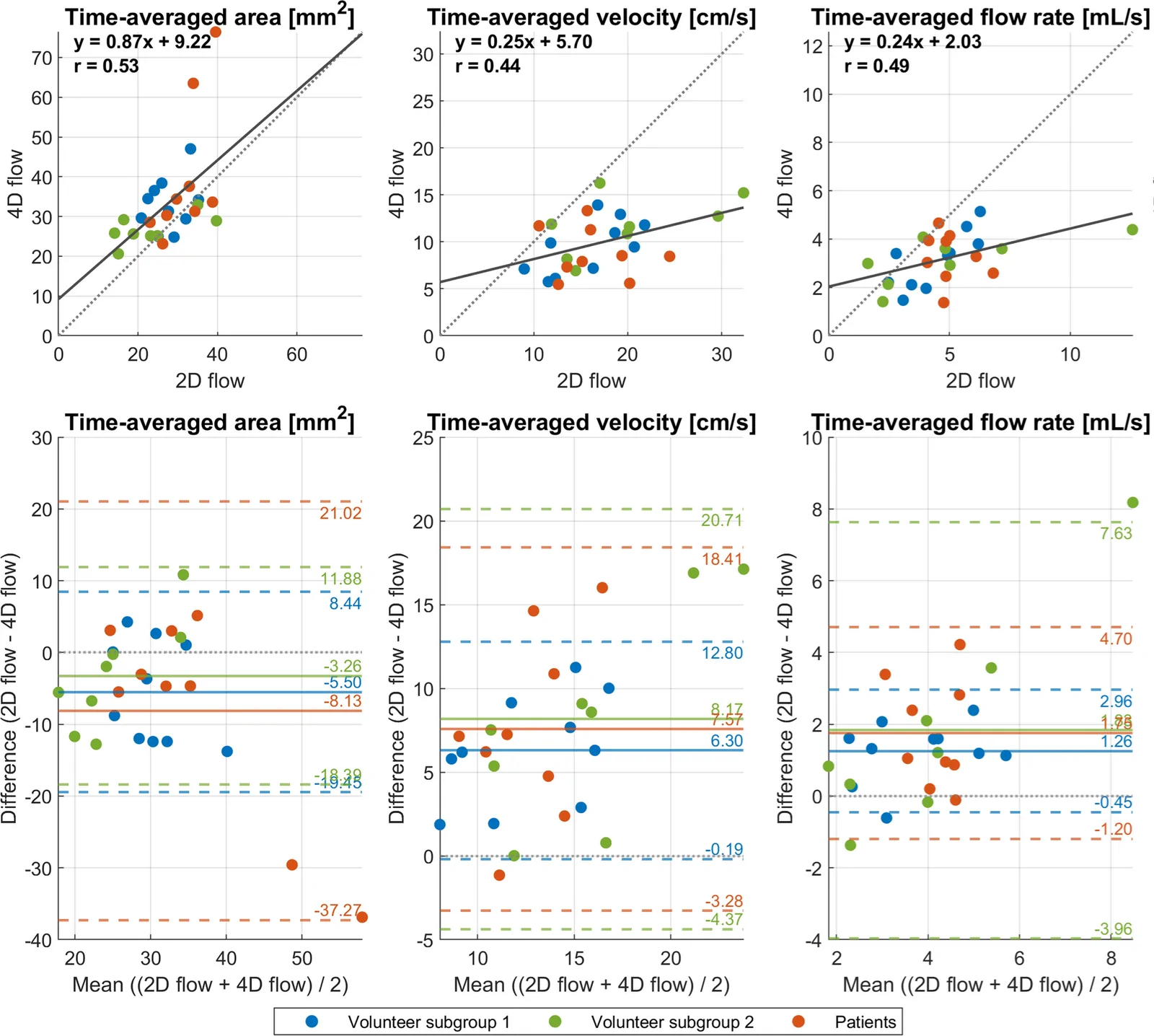
Analysis of agreement between 2D flow and 4D flow MRI-derived time-averaged hemodynamic parameters. Top row: Correlation plots of time-averaged hemodynamic parameters from 2D and 4D flow acquisitions. The dotted line indicates the identity line (y = x). Solid lines represent linear regression fits, with corresponding formulas and Pearson’s correlation coefficients (*r*) shown in each plot. Bottom row: Bland–Altman plots of the same data. The dotted grey lines represent the zero-difference lines (y = 0); solid lines indicate the mean difference, and dashed lines represent the limits of agreement

Agreement between 2D and 4D flow measurements was assessed using Pearson’s correlation and Bland–Altman analysis. Two subjects were excluded due to suboptimal 2D flow plane orientation. The results are presented in Table 5, Fig. 4 (time-averaged parameters), and Supplementary Fig. S1 (peak systolic parameters). Pearson’s correlation coefficients ranged from *r* = 0.44 to *r* = 0.54 across parameters, indicating moderate linear relationships between the two modalities. Area_avg_ and area_peak_ were larger in the 4D flow than in 2D flow measurements, on average by 5.7 mm^2^ (17.5%) and 5.8 mm^2^ (18.2%), respectively. In contrast, v_avg_ and v_peak_ were on average smaller in the 4D flow than in the 2D flow measurements, respectively by 7.3 cm/s (52.5%) and 16.3 cm/s (52.9%). This resulted in an overall underestimation of flow_avg_ and flow_peak_ in 4D flow measurements, by respective values of 1.6 mL/s (36.5%) and 3.4 mL/s (35.3%).

**Table 5:** Results of the 2D flow versus 4D flow MRI-derived hemodynamic parameters

	Mean difference	Lower LOA	Upper LOA	Pearson’s correlation coefficient
area_avg_	− 5.7 mm^2^	− 25.8 mm^2^	14.4 mm^2^	0.53
area_peak_	− 5.8 mm^2^	− 25.6 mm^2^	14.0 mm^2^	0.54
v_avg_	7.3 cm/s	− 2.5 cm/s	17.1 cm/s	0.44
v_peak_	16.3 cm/s	− 6.5 cm/s	39.1 cm/s	0.45
flow_avg_	1.6 mL/s	− 2.0 mL/s	5.2 mL/s	0.49
flow_peak_	3.4 mL/s	− 4.6 mL/s	11.4 mL/s	0.51

*LOA* limit of agreement (calculated as bias ± 1.96 × standard deviation), *area*_*avg*_ time-averaged area, *area*_*peak*_ peak systolic area, *v*_*avg*_ time-averaged velocity, *v*_*peak*_ peak systolic velocity, *flow*_*avg*_ time-averaged flow rate, *flow*_*peak*_ peak systolic flow rate

**Fig. 5:**
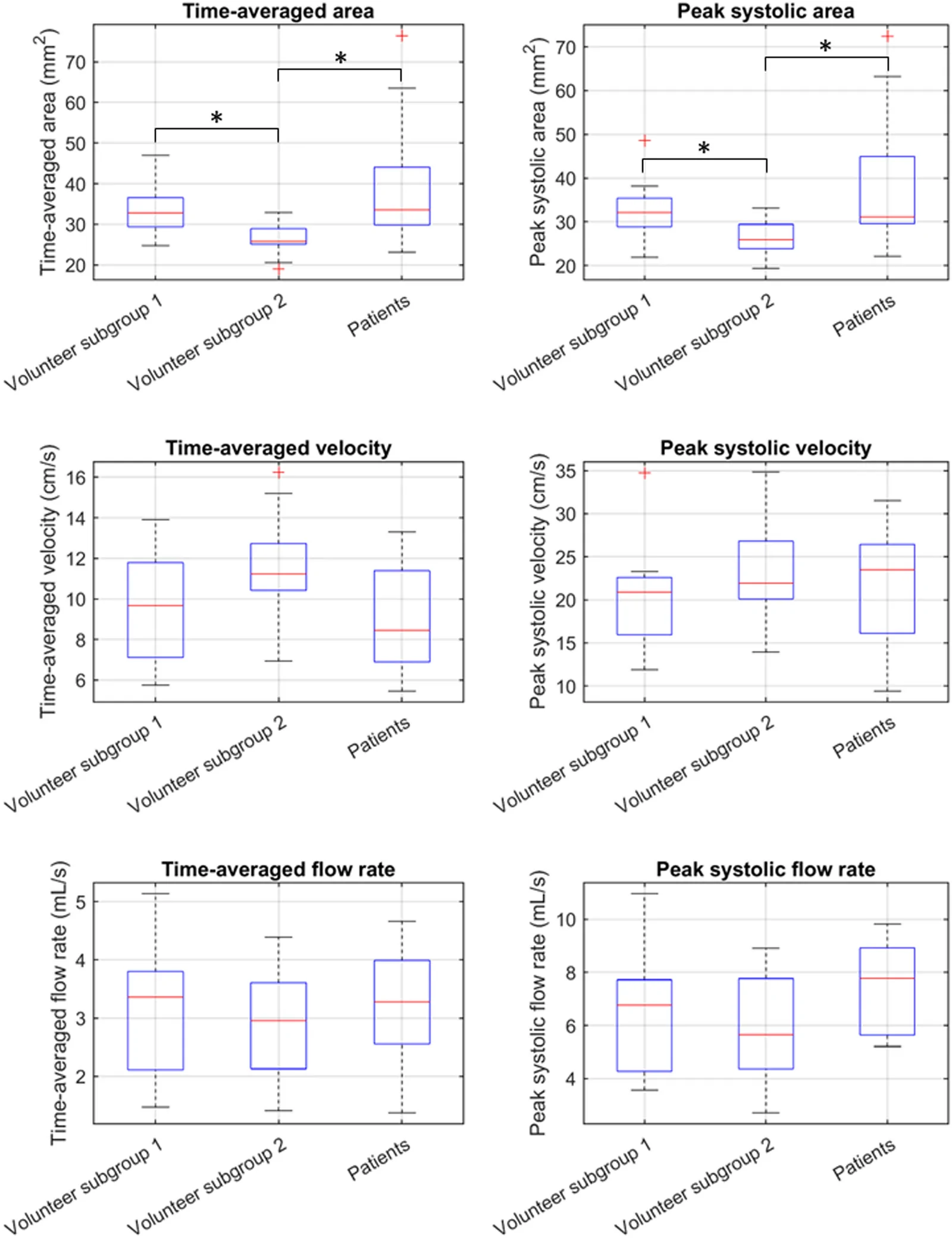
Comparison of 4D flow MRI-derived hemodynamic parameters between the three subgroups. Time-averaged area and peak systolic area were significantly lower in volunteer subgroup 2 compared to both volunteer subgroup 1 and the patient subgroup (*p* < 0.05). Significant differences are indicated in the boxplots with an asterisk (*). No other significant differences were found. Red crosses indicate outliers, defined as data points located more than three times the interquartile range below the first quartile or above the third quartile

Exploratory subgroup comparisons of 4D flow-derived hemodynamic parameters (acquisition 1, proximal plane) revealed significantly lower area_avg_ and area_peak_ in volunteer subgroup 2 compared to both volunteer subgroup 1 and the patient subgroup (Table 2, Fig. 5). No other significant differences were observed between subgroups.

## Discussion

Feasibility rates in healthy volunteers matched those reported by Dimov et al. [10], and feasibility rates in patients aligned with findings by Bane et al. [15]. Compared to the healthy volunteers reported by Dimov et al. [10], our flow_avg_ values in volunteer subgroup 1 were similar (~ 3 mL/s). However, our area_avg_ was ~ 27% larger (~ 33 mm^2^ versus ~ 26 mm^2^), while v_avg_ and v_peak_ were ~ 60% and ~ 48% lower, respectively (~ 10 cm/s versus ~ 25 cm/s and ~ 21 cm/s versus ~ 40 cm/s, respectively). This may be attributed to their lower VENC (40 cm/s), enhancing MR signal in the CHA, but at a risk of aliasing.

Consistent with our findings, Dimov et al. [10] reported that 4D flow overestimates areas and underestimates velocities and flow rates compared to 2D flow. However, their 2D and 4D flow sequences differed in spatial resolution and VENC, whereas ours were matched. Underestimation of HABF by 4D flow MRI can be attributed to systematic velocity underestimation and the indirect effect of vessel area overestimation, as larger segmentations include peripheral pixels with slower flow, potentially due to respiratory motion artifacts. Moreover, lower temporal resolution and fewer cardiac phases in the 4D flow acquisition, trade-offs to enable full volumetric coverage within a clinically acceptable scan time, explain the lower velocities because peak velocities were not accurately detected, as shown in Fig. 2. Software differences [16, 17], CS acceleration [18–20], and cardiac gating errors from magnetohydrodynamic interference [21] may also contribute to velocity underestimation of 4D flow. Thus, systematic differences between 2D and 4D flow-derived hemodynamic parameters should be considered when interpreting results, and comparisons should be made with caution.

Stankovic et al. [22] previously investigated scan-rescan repeatability of the HABF parameters area_avg_, v_avg_, v_peak_, and flow_avg_. We observed better repeatability across all parameters, except for v_peak_. Our improved repeatability across the majority of parameters could be attributed to our shorter scan-rescan time interval, subject-specific VENC approach, increased spatial and temporal resolution, and higher number of cardiac phases. In contrast to Stankovic et al. [22], we observed that average-derived parameters were generally more stable across repeated acquisitions, likely due to the increased signal-to-noise ratio (SNR) from averaging and reduced sensitivity to cardiac gating errors and physiological fluctuations.

Several factors may have contributed to the remaining within-subject variability, including the relatively large respiratory gating window, suboptimal cardiac gating, small vessel diameter relative to voxel size, and physiological variation between scans. On average, we did, however, comply with the 5 voxels per vessel diameter rule stated in literature [12]. Inter- and intra-observer variability are expected to contribute to the remaining variability as well, but were not assessed in the current study. Bane et al. [15] evaluated inter-observer agreement of 4D flow-derived hemodynamic parameters obtained from the CHA in ten patients with portal hypertension, and found CVs of 11% for area_avg_, 20% for v_avg_, 21% for v_peak_, and 19% for flow_avg_, approximating half our CV_total_. Although this provides an indication of the contribution of observer-related variability to total variability, the results cannot be directly translated to our findings due to differences in acquisition protocols.

A key limitation of this study is the lack of inter- and intra-observer variability assessment, which may affect the objectivity and reproducibility of the findings. Future studies should address this by including repeated measurements by the same and multiple observers. Moreover, this study focused on patients with cirrhosis and/or hepatic malignancy due to their relevance to TARE planning. Future studies may include a broader spectrum of liver pathologies to enhance generalisability. In addition, the ability to characterise HABF may also be relevant for other intra-arterial therapies, such as chemoembolisation or bland embolisation, where flow dynamics can influence treatment distribution and efficacy.

A major advantage of the 4D flow acquisition used in this study is its relatively high spatial resolution, improving vessel visibility and enabling more accurate segmentation. However, this comes at the cost of reduced SNR. Nevertheless, as previously mentioned, repeatability in our study exceeded that reported in earlier work [22].

Despite the use of CS acceleration, the mean acquisition time of our 4D flow sequence remained relatively long at 13 min. This duration does not include the additional time required for selecting the appropriate VENC, which can add several more minutes. Such extended acquisition times pose a challenge for routine clinical implementation, where efficiency and patient throughput are critical. To address this limitation, ongoing research is exploring advanced acquisition strategies aimed at reducing 4D flow acquisition times [15, 23].

We emphasise that this study only assessed repeatability, not accuracy. While prior phantom studies have addressed accuracy [10], clinical in vivo validation remains limited due to the absence of a gold standard.

Although 4D flow MRI requires longer acquisition than 2D flow MRI, it offers several advantages and potential clinical values. The first and most important benefit is the ability to retrospectively position measurement planes, which is particularly relevant in the hepatic artery. Accurate plane positioning is essential for reliable flow measurements, yet it can be challenging due to anatomical variations, vessel tortuosity, respiratory motion, and small vessel size. Second, the 4D flow acquisition utilised in the current study does not require beath-holding, which is beneficial for patients with compromised respiratory function. Third, in the field of computational fluid dynamics to study TARE [24], 4D flow MRI can provide realistic boundary conditions, thereby improving simulation accuracy. Fourth, in the context of MRI-guided TARE [25], 4D flow MRI can provide patient-specific insights into hepatic arterial hemodynamics. This could help clinicians with treatment planning, for example by comparing HABF at various catheter positions and adjusting the microsphere injection technique accordingly [6]. This requires higher signal in distal branches of the hepatic artery, which could be achieved by applying a lower VENC, for example the 40 cm/s utilised by Dimov et al. [10]. Intraprocedural HABF measurements could also be valuable, as changes may occur due to catheter positioning or microsphere injection. However, this requires the technique to be sensitive and robust enough to detect such (potentially small) changes in HABF, warranting further investigation. Moreover, it is crucial to understand which HABF variations meaningfully affect microsphere distribution. In our previous work, average HABF rates of 7.3 ± 1.4 mL/s (~ 37.3 ± 7.2 cm/s) yielded different microsphere distribution patterns compared to average HABF rates of 9.7 ± 0.25 mL/s (~ 49.5 ± 1.3 cm/s) [6]. These differences in both flow and velocity exceed the scan-rescan variability observed in the current study (SD_within_: 0.6 mL/s for flow_avg_, 1.8 cm/s for v_avg_), indicating that these variations are reliably detectable.

To optimise 4D flow MRI for clinical use, we recommend lowering the VENC to improve the velocity-to-noise ratio and segmentation robustness. Aliasing correction techniques should be applied to mitigate associated risks. Dual- or multi-VENC strategies may extend the measurable velocity range and improve accuracy [26]. Additionally, narrowing the respiratory acceptance window could reduce motion artifacts, though this is currently not recommended due to the already prolonged acquisition time. Finally, the implementation of more robust cardiac gating techniques, particularly those less affected by magnetohydrodynamic interference, which is amplified at 3T, should be explored [21]. Imaging at 1.5T reduces this interference but at the cost of SNR, potentially compromising image quality.

In conclusion, quantifying HABF using 4D flow MRI is feasible, with image quality sufficient in the majority of cases. However, substantial scan-rescan variability exists, as reflected by coefficients of variation of up to 30%. Acquisition-related factors accounted for the largest proportion of within-subject variability (30–40%), while plane positioning contributed a smaller portion (7–11%). These findings underscore the need for future studies to develop strategies that reduce or compensate for these sources of variability.

## Supplementary Information

Below is the link to the electronic supplementary material.

Supplementary file1 (DOCX 316 KB)

## Data Availability

The datasets generated and/or analysed during the current study are available from the corresponding author on reasonable request.

## Abbreviations

2D: Two-dimensional
2D flow MRI: Two-dimensional time-resolved phase-contrast MRI with through-plane velocity encoding
4D: Four-dimensional
4D flow MRI: Three-dimensional time-resolved phase-contrast MRI with three-dimensional velocity encoding
area_avg_: Time-averaged area
area_peak_: Peak systolic area
BMI: Body mass index
CHA: Common hepatic artery
CS: Compressed sensing
CV: Coefficient of variation
CV_total_: Total coefficient of variation
CV_within_: Within-subject coefficient of variation
flow_avg_: Time-averaged flow rate
flow_peak_: Peak systolic flow rate
HABF: Hepatic arterial blood flow
HCC: Hepatocellular carcinoma
ICC: Intraclass correlation coefficient
ICC_between_: Intraclass correlation coefficient for between-subject variance
ICC_within_: Intraclass correlation coefficient for within-subject variance
ICC_within, acquisition_: Intraclass correlation coefficient for within-subject variance due to acquisition
ICC_within, plane_: Intraclass correlation coefficient for within-subject variance due to plane positioning
ICC_within, overall_: Combined within-subject intraclass correlation coefficient (acquisition + plane)
LOA: Limits of agreement
MRI: Magnetic resonance imaging
PC-MRA: Phase-contrast magnetic resonance angiogram
RAVE: Radial volumetric encoding
REML: Restricted maximum likelihood
SD: Standard deviation
SD_total_: Total standard deviation
SD_within_: Within-subject standard deviation
T2*w: T2*-weighted
TA: Acquisition time
TARE: Transarterial radioembolisation
TE: Echo time
TR: Repetition time
TRUFI: True fast imaging with steady-state free precession
v_avg_: Time-averaged velocity
VCA: Variance components analysis
VENC: Velocity encoding limit
VIBE: Volumetric interpolated breath-hold examination
v_peak_: Peak systolic velocity
