# Supplementary material for: Quantifying hepatic arterial blood flow with 4D flow MRI: an in vivo feasibility and repeatability study
Source: MAGMA. 2026 Feb 14;39(5):709–22. doi: 10.1007/s10334-026-01331-0 (PMC13719827; doi:10.1007/s10334-026-01331-0)
Supplement: Supplementary file 1 [file 10334_2026_1331_MOESM1_ESM.docx]

| **Pt. #** | **Age [years]** | **Sex** | **Liver disease** | **Lesion(s) present** | **Etiology** | **Child-Pugh score** | **Lesions size, location and LI-RADS score (if applicable)** | **Prior treatment** |
| --- | --- | --- | --- | --- | --- | --- | --- | --- |
| **1** | 77 | M | Cirrhosis + HCC | Yes | MetALD | A | 35 mm (segment V/VI) LI-RADS 5, 24 mm (segment III) LI-RADS 5, 13 mm (segment VI) LI-RADS 4 | - |
| **2** | 59 | M | FLD with focal steatosis | - | - | - | - | - |
| **3** | 61 | M | CRLM | No^†^ | - | - | - | Multiple lines of chemotherapy, resection segment IVa |
| **4** | 25 | M | Cirrhosis | Yes | ARPKD | A | 8 mm (segment I) LI-RADS 2 | - |
| **5** | 79 | F | CRLM | No^†^ | - | - | - | MWA segment V, VI and VIII |
| **6** | 68 | M | Cirrhosis | No | AIH | A | - | - |
| **7** | 43 | F | Cirrhosis | Yes | AIH | A | 9 mm (segment IV/VIII) LI-RADS 3, 16 mm (segment IVA) LI-RADS 3 | - |
| **8** | 73 | M | Cirrhosis + HCC | Yes | MetALD | B | 16 mm (segment IV/VIII) LI-RADS 5, 12 mm (segment VIII) LI-RADS 4, 8 mm (segment III) LI-RADS 3 | - |
| **9** | 68 | M | Cirrhosis | Yes | MASLD | B | 8 mm (segment IV) LI-RADS 3 | - |

***Supplementary Table S1*** *Patient characteristics Pt. # = patient number, LI-RADS = liver imaging reporting and data system, HCC = hepatocellular carcinoma, MetALD = metabolic dysfunction and alcohol associated liver disease, FLD = fatty liver disease, CRLM = colorectal liver metastasis, ARPKD = autosomal recessive polycystic kidney disease, MWA = microwave ablation, AIH = autoimmune hepatitis, MASLD = metabolic dysfunction-associated steatotic liver disease. ^†^ No more lesion(s) present due to treatment response.*

***Supplementary Figure S1*** *Analysis of agreement between 2D flow and 4D flow-derived peak systolic hemodynamic parameters. Top row: Correlation plots of peak systolic hemodynamic parameters from 2D and 4D flow acquisitions. The dotted line indicates the identity line (y = x). Solid lines represent linear regression fits, with corresponding formulas and Pearson’s correlation coefficients (r) shown in each plot. Bottom row: Bland-Altman plots of the same data. The dotted grey lines represent the zero-difference lines (y = 0); solid lines indicate the mean difference, and dashed lines represent the limits of agreement.*
